# Association Between Electroencephalogram-Derived Sleep Measures and the Change of Emotional Status Analyzed Using Voice Patterns: Observational Pilot Study

**DOI:** 10.2196/16880

**Published:** 2020-06-09

**Authors:** Hirotaka Miyashita, Mitsuteru Nakamura, Akiko Kishi Svensson, Masahiro Nakamura, Shinichi Tokuno, Ung-Il Chung, Thomas Svensson

**Affiliations:** 1 Precision Health, Department of Bioengineering Graduate School of Engineering University of Tokyo Tokyo Japan; 2 Internal Medicine Mount Sinai Beth Israel New York, NY United States; 3 Voice Analysis of Pathophysiology Graduate School of Medicine University of Tokyo Tokyo Japan; 4 Department of Clinical Sciences Lund University Skåne University Hospital Malmö Sweden; 5 Department of Diabetes and Metabolic Diseases University of Tokyo Tokyo Japan; 6 School of Health Innovation Kanagawa University of Human Services Kawasaki-ku, Kawasaki-shi, Kanagawa Japan; 7 Clinical Biotechnology, Center for Disease Biology and Integrative Medicine Graduate School of Medicine University of Tokyo Tokyo Japan

**Keywords:** voice analysis, emotional status, vitality, sleep, mobile phone

## Abstract

**Background:**

Measuring emotional status objectively is challenging, but voice pattern analysis has been reported to be useful in the study of emotion.

**Objective:**

The purpose of this pilot study was to investigate the association between specific sleep measures and the change of emotional status based on voice patterns measured before and after nighttime sleep.

**Methods:**

A total of 20 volunteers were recruited. Their objective sleep measures were obtained using a portable single-channel electroencephalogram system, and their emotional status was assessed using MIMOSYS, a smartphone app analyzing voice patterns. The study analyzed 73 sleep episodes from 18 participants for the association between the change of emotional status following nighttime sleep (Δvitality) and specific sleep measures.

**Results:**

A significant association was identified between total sleep time and Δvitality (regression coefficient: 0.036, *P*=.008). A significant inverse association was also found between sleep onset latency and Δvitality (regression coefficient: –0.026, *P*=.001). There was no significant association between Δvitality and sleep efficiency or number of awakenings.

**Conclusions:**

Total sleep time and sleep onset latency are significantly associated with Δvitality, which indicates a change of emotional status following nighttime sleep. This is the first study to report the association between the emotional status assessed using voice pattern and specific sleep measures.

## Introduction

The evaluation of emotion is a challenge in many studies. Questionnaires have been the gold standard to evaluate emotion [1], but they cannot avoid reporting bias [2]. To solve this problem, a novel approach to analyze emotional status has been developed: Voice analysis [3], which analyzes the acoustic features of voice to estimate the involuntary effect of autonomic nerves on vocal cord movement and calculates vitality as a measure of mental status [4]. Primarily, high vitality could be translated into positive moods, as Hagiwara et al [4] revealed an inverse association between vitality index and the Beck Depression Inventory. Vitality can be used to evaluate the therapeutic effect of continuous positive airway pressure as a treatment of sleep apnea syndrome [5].

Sleep is one of the essential activities for humans. Extensive studies have been conducted to elucidate the relationship between sleep and affect [6]. A systematic review by Konjarski et al [7] revealed the reciprocal relationship between positive mood and better quality, shorter latency, and regular duration of sleep. There is also some evidence on the association between sleep loss and emotion in a general population [8], albeit with some discrepancies between research articles.

Previous studies have revealed that sleep deprivation is associated with altered autonomic nervous activities [9,10]. The vocal cord is partially innervated with autonomic nerves—the superior laryngeal nerve and the recurrent laryngeal nerve. Therefore, it can be hypothesized that sleep insufficiency may negatively affect voice quality, and further that sleep may influence vitality. Good sleep may be associated with the change in vitality from evening to morning (Δvitality), given, based on previous studies, that good sleep improves mood positively, which in turn might bring about the increment of vitality overnight. Therefore, it was hypothesized that an appropriate duration of sleep, 7-9 hours for adults [11], is associated with a greater recovery of vitality, that is, a higher Δvitality.

## Methods

### Participants

The participants were 20 healthy Japanese adults (10 men and 10 women; age 25-67 years) recruited from Kanagawa and Tokyo. For the purpose of this study, only participants with repeated measures of the outcome variable (n=18, described in detail later) were included.

Participation in the study was entirely voluntary, and all participants were informed about the purpose of the study and that participation could be discontinued at any point without any disadvantage or imposed penalty for the participants. All research was performed in accordance with relevant guidelines/regulations. All participants provided written informed consent, and the study was approved by the Ethical Committee of the Department of Bioengineering, Graduate School of Engineering, the University of Tokyo (Approval number: KE18-7).

### Study Design

This is an observational pilot study to investigate the association between electroencephalogram (EEG)-derived sleep measures and the change of emotional status analyzed from voice. The aim of the overall study from which the data were obtained was to validate the sleep-tracking functions of a consumer wearable device against a validated portable single-channel EEG system in naturalistic conditions. Details of the validation study can be found in the study by Svensson et al [12]. No restrictions for sleep or activity were imposed on the participants. Sleep measures in this study were obtained using the validated portable single-channel EEG system (SleepScope, SleepWell Co, Ltd.; the device is described in greater detail later). Participants were asked to use the device every night throughout the study period. Participants were also provided with an internet-connected Android smartphone, which had preinstalled an app (MIMOSYS; the app is described in greater detail later) that utilizes voice analysis for assessing overall mental wellness. During the study period, participants were asked to use MIMOSYS twice per day to obtain morning and evening vitality measures.

Sleep data were available for a total of 7 nights, equivalent to a total of 138 sleep episodes (person-nights). Details of sleep data retrieval and retention are described by Svensson et al [12].

### Exposures

The main exposure of this study was total sleep time (minutes) converted into sleep hours (total sleep time/60). Total sleep time was obtained using EEG. Details of the single-channel EEG system can be found in the validation study by Yoshida et al [13]. In brief, the portable single-channel EEG system (SleepScope) is manufactured by SleepWell Co, Ltd. and is a registered medical device (Japanese Medical Device Certification number: 225ADBZX00020000) suitable for home use. The SleepScope has shown acceptable agreement with polysomnography and records waveforms appropriate for sleep staging (wake [W], R, N1, N2, and N3) as defined by version 2.3 of the American Academy of Sleep Medicine Scoring Manual [14].

Secondary endpoints (sleep efficiency, sleep onset latency, and number of awakenings during sleep) were chosen on the basis of a meta-analysis conducted by Baglioni et al [15]. This meta-analysis revealed that a problem in sleep continuity exists in most psychiatric disorders and that total sleep time, sleep efficiency, sleep onset latency, and number of awakenings are components of sleep continuity. Sleep efficiency was defined as the ratio of total sleep time to time in bed × 100 (%). Sleep onset latency was the time from going to bed to sleep onset. Number of awakenings was defined as the number of continuous records of W after sleep onset.

### Vitality

The main outcome measure using the MIMOSYS voice analysis software was the change in vitality score from evening to morning (ie, Δvitality calculated as morning vitality – evening vitality). The algorithm of the MIMOSYS was originally developed to enable screening of major depression using only voice analysis. The basic idea of MIMOSYS is to check for continuously depressed mood, one of the diagnostic criteria for major depression [16]. The software is developed and provided by PST Inc. In the algorithm, vitality was derived from proportions of elemental emotions evaluated from single utterance delimited by breathing using the Sensibility Technology Software Development Kit (PST Inc). The vitality for a single measurement was acquired by averaging vitality acquired from sequential utterances within the same measurement. This study used an app implemented for Android OS with additional functions such as voice recording, monitoring of execution status for each participant, and questionnaires. The app was installed on smartphones (ZenFone 2 Laser, ASUSTeK Computer Inc), which were provided to each participant of the study.

**Figure 1:**
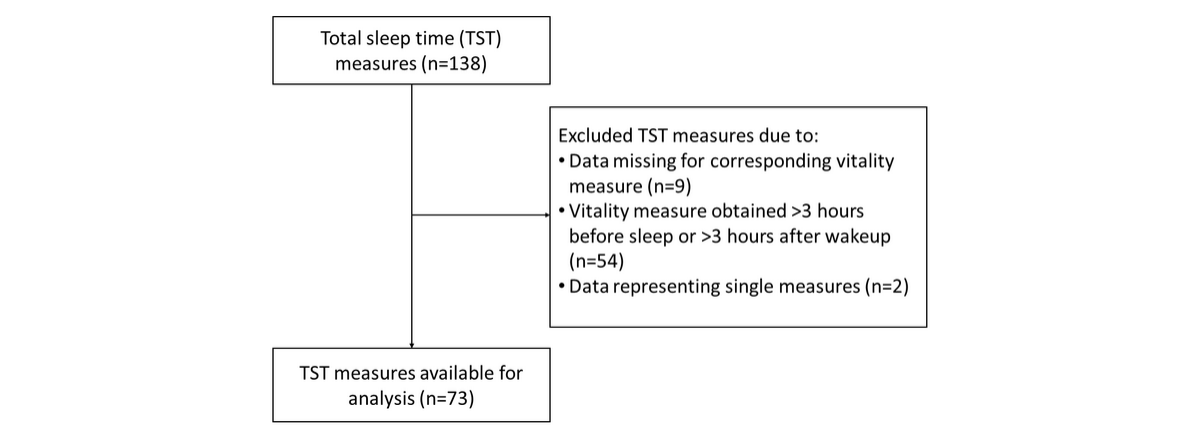
Flowchart of participants included in the study.

During the study period, there were 138 measures of evening vitality and 137 measures of morning vitality (Figure 1). One evening measure was excluded, as it would not allow for the calculation of Δvitality. In addition, 8 sleep measures were excluded, as they did not have a corresponding morning vitality measure (ie, the participant did not use the MIMOSYS app in the morning). A total of 54 sleep measures were excluded, as there were no corresponding vitality measures in the 3 hours preceding sleep or in the 3 hours following awakening as determined by EEG. Finally, the sole observations of two participants were excluded as the chosen statistical methods focus on repeated measures analysis. Consequently, there were 73 person-nights’ data of sleep from 18 participants with corresponding Δvitality measures available for analysis.

### Statistical Analyses

A total of 18 participants with at least two measures (range, 2-7 measures) of sleep hours with a corresponding value of Δvitality for the sleep period were included in the analysis. Generalized estimating equations (GEEs) were used to estimate the change in vitality score from evening to morning as a function of the exposure. Given the normally distributed outcome measure (Δvitality), the GEE model family was set as *gaussian,* with the link function specified as *identity,* the correlation structure specified as *unstructured,* and the variance-covariance matrix of the estimators specified as *robust.* The analyses on the associations between total sleep time, sleep efficiency, sleep onset latency, and number of awakenings and Δvitality were conducted in separate statistical models. Models investigating the association of Δvitality with total sleep time and sleep onset latency were adjusted for age, sex, and evening vitality score. Models investigating the association between Δvitality and sleep efficiency and number of awakenings were additionally adjusted for total sleep time. All statistical analyses were performed using Stata/MP version 15.1 (StataCorp LLC). *P* values were two-tailed and considered significant if <.05.

### Data Availability Statement

We cannot publicly provide individual data due to participant privacy, according to ethical guidelines in Japan. Additionally, the written informed consent we obtained from study participants does not include a provision for publicly sharing data. Qualifying researchers may apply to access a minimal dataset by contacting the Principal Investigator for this study (TS).

TS had full access to all the data in the study and takes responsibility for the integrity of the data and the accuracy of the data analysis.

## Results

### Participants Characteristics

Participants’ baseline characteristics are summarized in Table 1. Of the 18 participants, 10 were male and the age of participants ranged from 25 to 67 years. The mean sleep duration was 5.4 (SD 1.23) hours. The means of sleep efficiency, sleep onset latency, and number of awakenings were 87.4%, 12.2 minutes, and 27.1 counts, respectively. The means of evening vitality, morning vitality, and Δvitality were 0.42, 0.37, and –0.05, respectively.

**Table 1:** Participants’ baseline characteristics.^a,b^

Variable	Value
Age (years), mean (SD); range	42 (11.8), 25-67
Male sex, n (%); range	10 (56), N/A^c^
Total sleep time (h), mean (SD); range	5.42 (1.23), 2.35-8.58
Sleep efficiency (%), mean (SD); range	87.36 (8.22), 49.2-95.8
Sleep onset latency (min), mean (SD); range	12.18 (15.39), 0.5-89.5
Number of awakenings, n (%); range	27.1 (12.35), 3-75
Evening vitality, mean (SD); range	0.42 (0.15), 0.004-0.85
Morning vitality, mean (SD); range	0.37 (0.13), 0.08-0.69
ΔVitality, mean (SD); range	–0.05 (0.17), –0.48 to 0.41

^a^N=18, which was the total number for age and male sex.

^b^N=73, which was the total number for the remaining variables.

^c^N/A: not applicable.

### Associations Between Exposures and ΔVitality

**Figure 2:**
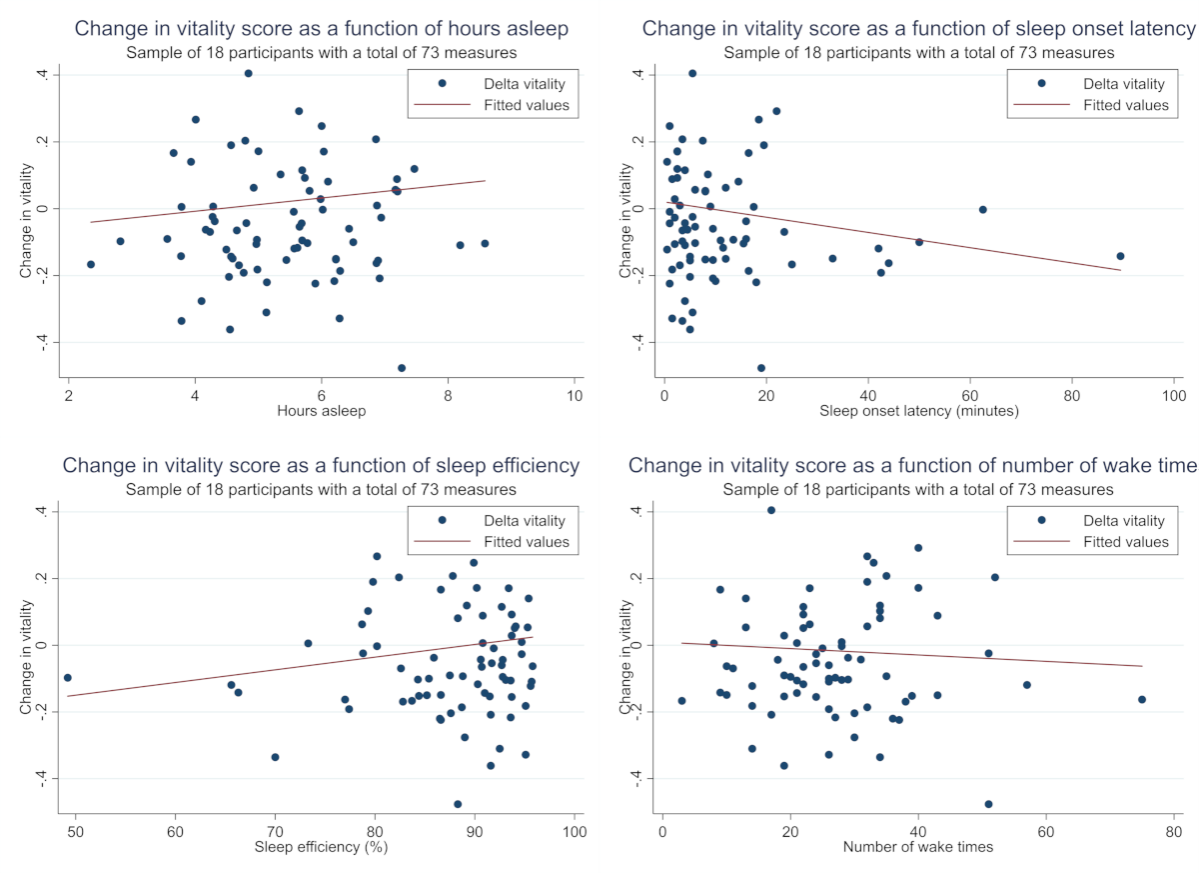
Scatter plots of exposures and Δvitality. The y-axis represents change of vitality before and after sleep. The x-axis represents each exposure (total sleep time, sleep onset latency, sleep efficiency, and number of awakenings). The solid line refers to fitted values. The closed circles refer to Δvitality.

When adjusted for age, sex, and evening vitality score, total sleep time showed a significant positive association with Δvitality (regression coefficient: 0.036, *P*=.008). A significant inverse association was also found between sleep onset latency and Δvitality (regression coefficient: –0.026, *P*=.001). The exposures sleep efficiency and number of awakenings showed no significant association with Δvitality even after additional adjustment for total sleep time (Table 2). The scatter plots expressing the exposures and Δvitality are shown in Figure 2.

**Table 2:** Regression coefficients for each exposure obtained from separate statistical models.

Exposure	Regression coefficient (95% CI)	*P* value
Total sleep time (hours)^a^	0.036 (0.0094 to 0.062)	.008
Sleep onset latency (min)^a^	–0.0026 (–0.0042 to –0.0010)	.001
Sleep efficiency (%)^b^	0.0022 (–0.0025 to 0.0069)	.36
Number of awakenings (n)^b^	0.00092 (–0.0015 to 0.0033)	.45

^a^Statistical model adjusted for age, sex, and evening vitality score.

^b^Statistical model adjusted for age, sex, evening vitality score, and total sleep time.

## Discussion

### Principal Findings

In this analysis of 73 measures of sleep from 18 adults, a significant positive association was found between total sleep time and Δvitality. Sleep onset latency was also found to be inversely associated with Δvitality. By contrast, there was no significant association between sleep efficiency and number of awakenings during sleep and Δvitality.

The magnitude of the regression coefficient (0.036) for the association between total sleep time and Δvitality is, despite its statistical significance, difficult to put into context given that there are no prior studies to compare with. As such, this is considered to be a pilot study with regard to the effect of sleep on the vitality measure obtained through voice analysis. The study hypothesis was that a sleep duration of 7-9 hours, which is considered appropriate for adults [11], is associated with a greater recovery of vitality, that is, a higher Δvitality. Indeed, the regression analyses indicated a statistically significant (*P*=.008) association between total sleep time and Δvitality, which is compatible with our hypothesis. There are some transient factors such as alcohol intake [17] that can affect voice patterns and, by extension, Δvitality. Although we did not adjust for these factors in study analyses, daily excessive alcohol intake was considered an exclusion criterion for participation in the study. The mechanisms for the found associations lie beyond the scope of this study; however, previous studies investigating the effect of sleep on the autonomic nervous system have found that short sleep duration on EEG is associated with low cardiac vagal regulation [18]. The result of this study also suggests that sleep duration is associated with autonomic nervous activity.

Very long sleep duration is known to be associated with adverse health outcomes [19], and sleeping for longer than 9 hours may be associated with psychiatric disorders [20]. In this study, the participants’ sleep durations ranged between 2.4 and 8.6 hours, with no participant recording a sleep duration longer than 9 hours. Consequently, no association between very long sleep duration and reduced Δvitality could be analyzed. Future studies are encouraged to investigate whether the recovery of vitality diminishes with excessive sleep duration.

Poor sleep continuity (low sleep efficiency, long sleep onset latency, and large number of awakenings) has been revealed to be associated with various psychiatric disorders. For example, Baglioni et al [15] showed statistically significant inferiority of sleep continuity in patients with affective disorders, anxiety disorders, and schizophrenia. Thus, it was hypothesized that sleep efficiency is positively, and sleep onset latency and number of awakenings are inversely associated with Δvitality. This study found an inverse association between sleep onset latency and Δvitality. This result suggests that a shorter sleep onset latency is associated with improved mental activity in the next morning. A previous article suggested that prolonged sleep onset latency is associated with autonomic nervous dysfunction as identified by reduced heart rate fluctuations during the sleep onset period [21]. This would be compatible with results of this study, given that the vocal cord is also partially controlled by the autonomic nervous system.

Sleep efficiency and number of awakenings are regarded as factors of sleep continuity and reported to be associated with some psychiatric disorders [15]. Furthermore, low sleep efficiency is reported to be associated with low levels of cardiac parasympathetic tone [18]. Thus, it was hypothesized that low sleep efficiency and large number of awakenings are associated with low Δvitality. However, an association between sleep efficiency or number of awakenings and Δvitality was not identified. This could be caused by actual null association; however, considering that the prior study was based on more than 500 measurements of sleep continuity [18], this study might have not had enough power to detect the association. In addition, the effect size of sleep efficiency and number of awakenings could be too small to override other unadjusted factors (eg, alcohol intake) contributory to Δvitality. More measurements and adjustments may be needed to address the association of Δvitality with sleep efficiency and number of awakenings.

MIMOSYS was used as a measure of mental status. The evaluation of mental status based on voice analysis is a new concept, whose validity should be carefully assessed. Currently, some evidence exists of voice analysis as a measure to assess mental status. Tokuno et al [22] showed that the voice analysis system had a sensitivity of 0.897 to detect patients with poor emotional hygiene who require medical intervention or counseling. Moreover, voice analyses had a comparable sensitivity when compared with the 30-item General Health Questionnaire (GHQ-30), a traditional questionnaire-based screening instrument to identify psychiatric conditions [23]. It was also reported that vitality determined from voice recordings is negatively correlated (–0.208) with the Beck Depression Inventory [4]. The study hypothesis was based on the assumption that voice patterns reflect mental status, which could be supported by these findings.

### Limitations

This study has a few limitations. First, not all data from MIMOSYS were utilized, because analyses in this study a priori excluded any vitality measures taken more than 3 hours before falling asleep or after awakening. However, this was done to capture the vitality measures on which sleep was believed to have the most influence. Any measures occurring several hours before or after a sleep period could be confounded by other lifestyle or environmental factors. Irrespectively, the measurement of vitality requires regular and repeated measurements by the participants, which may render the study susceptible to reporting bias. Second, a single-channel EEG system was used in this study to obtain sleep measures. The current gold standard of sleep monitoring is polysomnography [24]. However, polysomnography requires the participants to sleep in a special examination room, which may affect the results [25]. The single-channel EEG system is a relatively new technology, and its validation may not be fully established for certain sleep parameters [26]. Portable EEGs can be used in research participants’ natural sleep environments, which in turn may result in increased effectiveness as compared with polysomnography. Third, none of the participants had a sleep duration longer than 8.6 hours. This could result in an underestimation of the association between long sleep duration and Δvitality. Fourth, there is no available reference value to determine a healthy Δvitality score. Consequently, it is difficult to ascertain the clinical relevance of the regression coefficients obtained in this study. The main aim of this study, however, was not to establish a clinical cutoff, but to investigate whether sleep parameters were associated with a change in vitality. Finally, although Hagiwara et al [4] showed a correlation between the absolute value of vitality and the Beck Depression Inventory, no assessment has been conducted regarding Δvitality and emotion or well-being. Further studies are thus warranted to validate the findings of this pilot study.

### Strengths

Despite some limitations, this study has a number of strengths. First, this is the first study to show an association between sleep parameters and vitality as recorded through voice, which has the potential of a brand-new marker of emotional conditions and recovery from sleep. Notably, this study focused on Δvitality, a new variable defined as the difference of vitality before and after sleep. Second, this is a naturalistic study that obtained sleep and vitality measures in free-living conditions. As such, the results may be more readily generalizable to a daily living environment. Third, this study has used GEEs to investigate the association between sleep measures and Δvitality. Owing to the repeated measures of both exposure and outcome available in the data set, this method allows for the maximum utilization of the available information of correlated measures. Lastly, MIMOSYS, the app used to obtain vitality measures through voice analysis, is inexpensive, thereby allowing the study design to be easily replicated in future studies.

### Conclusion

A positive association was identified between total sleep time and Δvitality and an inverse association between sleep onset latency and Δvitality. These results suggest that specific sleep parameters are associated with the overnight change of vitality.

## Abbreviations

EEG: electroencephalogram
GEE: generalized estimating equation
GHQ-30: 30-item General Health Questionnaire
